# Histoplasma capsulatum Prosthetic Valve Endocarditis Diagnosed via Cell-Free DNA Sequencing

**DOI:** 10.7759/cureus.83292

**Published:** 2025-05-01

**Authors:** Aditya Patel, Shawn Doss, Gowtham Anche, Irielle Duncan, Daniel Anderson, Patrick Yue, Ashley Huggett

**Affiliations:** 1 Division of Infectious Diseases, Augusta University Medical College of Georgia, Augusta, USA; 2 Department of Internal Medicine, Touro College of Osteopathic Medicine, New York, USA

**Keywords:** diagnostic delay, fungal endocarditis, histoplasma capsulatum, karius test, microbial cell-free dna, next-generation dna sequencing, prosthetic valve infective endocarditis

## Abstract

*Histoplasma capsulatum *(*H. capsulatum*), a dimorphic fungus endemic to the Mississippi and Ohio River Valleys, is a rare cause of fungal infective endocarditis. Here, we present a case of prosthetic valve endocarditis due to *H. capsulatum* diagnosed via microbial cell-free DNA (cfDNA) sequencing when traditional methods, such as urine antigen and blood cultures, fail. A 56-year-old male patient with a history of a bioprosthetic mitral valve replacement presented with chest pain, confusion, and constitutional symptoms. Initial laboratory results, negative blood cultures, and echocardiographic findings were consistent with a diagnosis of culture-negative endocarditis. To identify a potential pathogen not detected by blood cultures, both urine *Histoplasma* antigen testing and the Karius Test®, a next-generation sequencing-based cfDNA assay, were performed concurrently. While traditional methods, including blood cultures and urine antigen testing, were negative, the Karius Test® identified *H. capsulatum*, prompting initiation of antifungal therapy. The patient subsequently showed clinical improvement, evidenced by the resolution of symptoms and a reduction in the size of the valvular lesion. This case highlights the diagnostic challenges of fungal endocarditis and underscores the utility of cfDNA sequencing in identifying elusive pathogens when conventional tests are inconclusive. Early recognition and targeted antifungal therapy are crucial for improving patient outcomes in culture-negative endocarditis cases.

## Introduction

Infective endocarditis (IE) is a rare but serious infection of the cardiac endothelium associated with high morbidity and mortality [1]. The most common causative organisms include *Staphylococcus aureus* and *viridans Streptococcus* species, with fungal pathogens accounting for only 1% to 3% of cases. Among fungal causes, *Candida* species (53% to 68% of patients) are the most common, followed by *Aspergillus* species (20% to 25%), *Histoplasma* species (~5%), and other fungi (~15%) [2]. *Histoplasma capsulatum *(*H. capsulatum*) is a dimorphic fungus endemic to the Mississippi and Ohio River Valleys, including West Virginia, where the patient had lived before relocating to Georgia. Common sources of exposure include caves and environments contaminated with bat or bird droppings. While typically asymptomatic or causing mild pulmonary disease, it can occasionally lead to disseminated infection, including endocarditis. Fewer than 100 cases of *Histoplasma* IE have been reported, with increased prevalence in individuals residing in endemic areas, those with valvular disease, and patients with prosthetic heart valves [2,3].

Diagnosis of *Histoplasma* IE traditionally involves *Histoplasma* antigen testing (urine or serum), serology, blood cultures, or histopathologic evaluation of affected tissues. However, *Histoplasma* endocarditis may be underdiagnosed in endemic regions like the Mississippi and Ohio River Valleys, where widespread latent or subclinical exposure can lead clinicians to overlook new infections, which is an issue further exacerbated by limited access to specialized diagnostics in the region’s rural and medically underserved communities. Furthermore, these methods can produce false-negative results in some cases due to a low organism burden or suboptimal timing of sample collection.

Next-generation sequencing of microbial cell-free DNA (cfDNA), exemplified by the Karius Test® developed in 2017, represents a novel diagnostic approach with high sensitivity and specificity, capable of detecting over 1,000 pathogens. As microorganisms turn over, they release fragments of DNA into the bloodstream. These cfDNA fragments are then analyzed using metagenomic sequencing combined with artificial intelligence to accurately identify the causative pathogen [4]. Here, we present a rare case of *H. capsulatum* prosthetic valve IE diagnosed using microbial cfDNA sequencing, despite negative urine *Histoplasma* antigen testing.

## Case presentation

A 56-year-old male patient presented to the emergency department at Wellstar Medical College of Georgia Health Medical Center with a chief complaint of chest pain, productive cough, and intermittent confusion over two weeks, with acute worsening the night prior to his arrival. His past medical history included rheumatic heart disease with mitral regurgitation and stenosis status post-bioprosthetic mitral valve replacement (Medtronic 29 mm) in 2020, atrial fibrillation treated with a biatrial maze procedure in 2019, heart failure with reduced ejection fraction (EF 45%), deep vein thrombosis status post-inferior vena cava filter placement in 2016, hypertension, depression, and dermatomyositis. Social history was notable for daily tobacco and alcohol use, with a remote history of cocaine use. The patient’s fiancée provided the majority of the history as the patient was lethargic and disoriented upon arrival; the information was later confirmed by the patient once he regained alertness and orientation.

On presentation, his vital signs were within reference limits. Significant laboratory findings included pancytopenia with elevated segmented neutrophils, alkaline phosphatase, aspartate aminotransferase, total bilirubin, and B-type natriuretic peptide, which may indicate cardiac strain (Table 1).

**Table 1:** Abnormal lab values

Test	Value	Reference range
Hemoglobin	13.3 g/dL	13.5-17.5 g/dL
Leukocytes	3,100/mm³	4500-11,000/mm³
Segmented neutrophils	79%	54%-62%
Platelets	107,000/mm³	150,000-400,000/mm³
Aspartate aminotransferase (AST)	45 U/L	12-38 U/L
Total bilirubin	1.6 mg/dL	0.1-1.0 mg/dL
B-type natriuretic peptide (BNP)	619 pg/mL	<100 pg/mL

Troponin was elevated at 0.143 ng/mL but quickly returned to normal levels (<0.04 ng/mL). A chest X-ray revealed bilateral hilar fullness without focal consolidations. Electrocardiogram (ECG) showed bigeminy without ST-segment elevations. Physical examination revealed an ill-appearing, lethargic patient with shallow breath sounds. Although the cardiac examination was normal, point-of-care cardiac ultrasound in the emergency department revealed a possible lesion on the bioprosthetic mitral valve.

**Figure 1:**
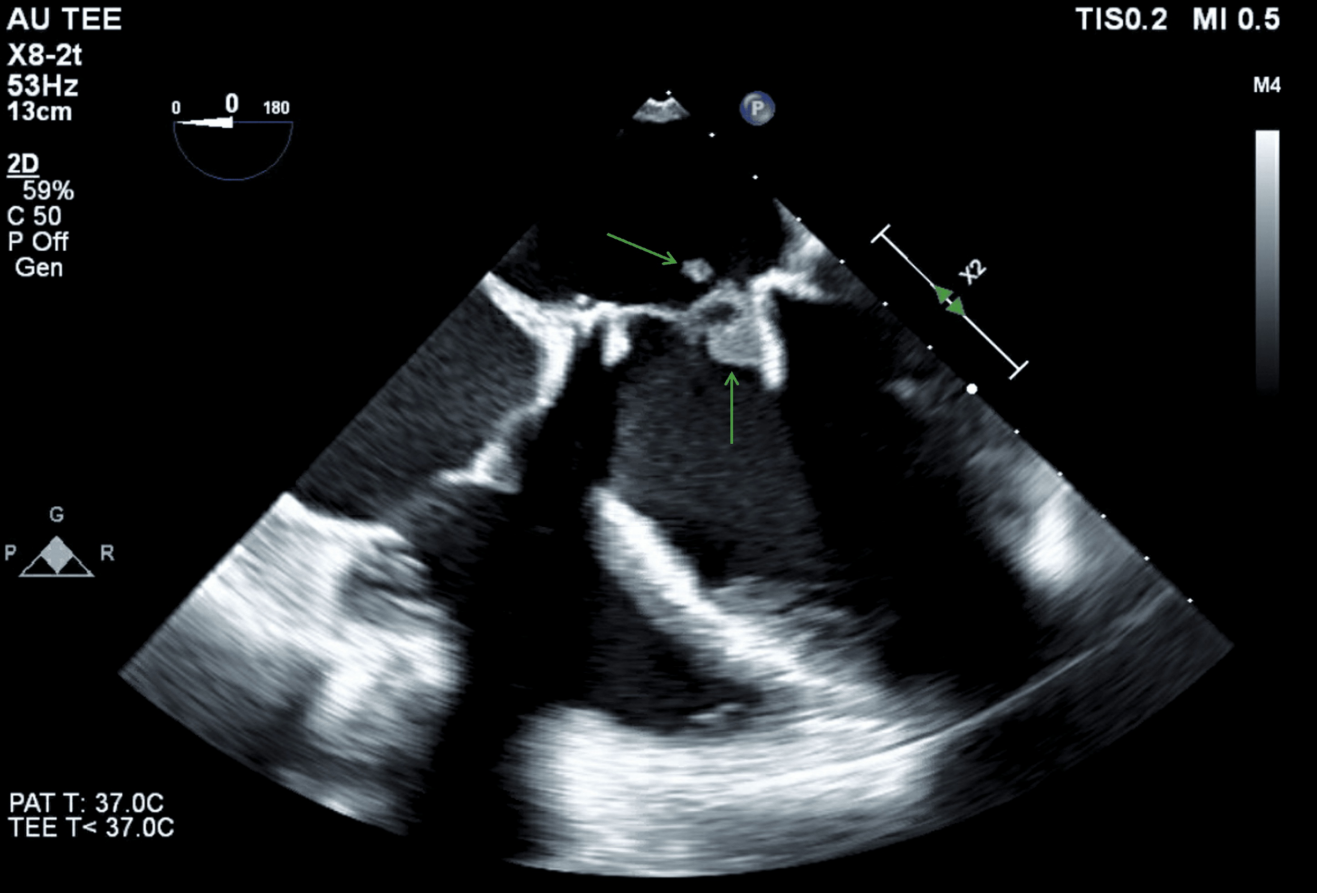
Transesophageal echocardiogram demonstrating a mobile hypodensity on the posterior leaflet and strut of the bioprosthetic Medtronic 29 mm mitral valve

Initial workup included blood cultures, urinalysis, serum lactic acid, rapid plasma reagin (RPR), B12, folate, human immunodeficiency virus (HIV), thyroid-stimulating hormone (TSH)/thyroxine (T4), ammonia, urine drug screen, and alcohol levels. Imaging included a noncontrast head computed tomography (CT), chest CT, and transthoracic echocardiogram (TTE). Empiric intravenous ceftriaxone and vancomycin were initiated for presumed endocarditis, and the patient was admitted to the internal medicine service. On admission, the laboratory results were notable only for a positive urine drug screen detecting amphetamines, cocaine, and tetrahydrocannabinol (THC). CT of the head and chest showed no abnormalities. Repeat blood cultures were drawn after the initiation of antibiotics. TTE revealed an EF of 47% with possible vegetations on the bioprosthetic mitral valve leaflets. The following day, a transesophageal echocardiogram (TEE) identified a small, mobile hypodensity on the posterior mitral valve prosthetic leaflet and the strut, consistent with vegetation; however, a thrombus could not be ruled out (Figure 1).

Cardiothoracic surgery was consulted and recommended medical management without surgical intervention to include a three-month course of warfarin for presumed thrombus, as well as repeating the TEE at the end of the treatment period to reassess the size of the lesion.

Infectious disease was also consulted. At the time of our evaluation, both sets of blood cultures showed no growth to date. A detailed social history was obtained to help elucidate the possible pathogen. The patient reported only three sexual partners. He did not report exposure to cave environments, bat or bird droppings, farm animals, pets, unpasteurized milk, sewage, recent sick contacts, international travel, air-conditioning units, cuts or injuries, incarceration, or homelessness. However, he worked as a garbage collector 40 years ago. He also lived in West Virginia for a few months but currently resides in Georgia.

Given the strong suspicion for culture-negative prosthetic valve endocarditis in the context of a negative diagnostic workup to date and lack of patient improvement, our recommendations included continuing intravenous vancomycin and ceftriaxone, repeating blood cultures, and obtaining urine testing for *Neisseria gonorrhoeae*, *Chlamydia trachomatis*, and *Histoplasma* antigen. A Karius Test® (microbial cell-free DNA via next-generation sequencing) was ordered concurrently to assist in pathogen identification, as the patient had not shown clinical improvement, and determining the causative organism was crucial for guiding management. As the causative pathogen had not yet been identified, serologic testing for specific organisms, including *H. capsulatum*, was deferred while awaiting the results of the Karius Test®.

Notably, while the vegetation may have represented a thrombus, empiric antibiotics were maintained pending further evaluation due to the potential risk of clinical deterioration if antimicrobial therapy was discontinued prematurely. Repeat blood cultures, urine gonorrhea/*Chlamydia* polymerase chain reaction (PCR), and urine *Histoplasma *antigen returned negative; however, Karius Test® was positive for *H. capsulatum* with a concentration of 209 molecules per microliter (MPM). In response, intravenous vancomycin and ceftriaxone were discontinued, and liposomal amphotericin B was initiated at 5 mg/kg/day.

The infectious disease team noted that while rare, a negative urine antigen test could occur in disseminated histoplasmosis when the infection is confined to a specific organ, such as the heart [5]. In such cases, tissue sampling may be necessary to establish a definitive diagnosis, which was not pursued due to the patient’s improving symptoms.

During his hospital stay, the patient remained pancytopenic. Given his normal complete blood count (CBC) from the prior year, hematology/oncology was consulted to investigate the possibility of bone marrow involvement by disseminated histoplasmosis. A bone marrow biopsy was performed, and samples were sent for flow cytometry, karyotyping, fluorescence in situ hybridization (FISH), acid-fast bacillus staining, Grocott’s methenamine silver (GMS) staining, and periodic acid-Schiff (PAS) staining. All results were negative for fungal infection, malignancy, or other hematologic abnormalities.

The patient received five days of liposomal amphotericin B before transitioning to oral itraconazole. He was loaded with itraconazole over three days and then maintained on 200 mg twice daily for a planned 12-week course. The duration of treatment with amphotericin B followed by itraconazole was guided by patient outcomes reported in prior case studies [3]. The duration of antifungal therapy beyond 12 weeks would be reassessed based on the residual size of the vegetation and the presence or absence of ongoing symptoms. Anticoagulation was transitioned from heparin to warfarin during hospitalization, with plans to continue warfarin alongside itraconazole after discharge, as both therapies had contributed to the patient’s clinical improvement. Despite persistent pancytopenia (unchanged from admission), the patient was stable and discharged with a scheduled TEE three months post-discharge and a follow-up with infectious disease in three weeks.

At his follow-up appointment with infectious disease, the patient was reportedly doing well. He had no complaints and was maintained on his itraconazole and warfarin. A repeat TEE showed a reduction in the size of the valvular lesion. Notably, although surgical intervention is generally recommended in most cases of prosthetic valve fungal endocarditis, the absence of severe valvular dysfunction and the patient’s favorable clinical response to targeted antifungal therapy supported a nonsurgical management approach at this time.

## Discussion

Fungal endocarditis is an exceptionally rare subtype of IE, comprising only 1% to 3% of cases. Within this subset, *Candida* species are the most frequently isolated pathogens, accounting for 53% to 68% of fungal endocarditis cases, followed by *Aspergillus* species at 20% to 25% [2,6]. Less common fungal pathogens include *H. capsulatum*, *Blastomyces dermatitidis*, *Coccidioides immitis*, and *Cryptococcus neoformans*. *H. capsulatum* endocarditis is particularly uncommon, with a systematic review identifying only 61 reported cases between 1940 and 2020 [7].

Several risk factors predispose patients to fungal endocarditis, including structural valvular dysfunction, prosthetic heart valves, prior episodes of IE, implanted cardiac devices, intravenous drug use, chronic kidney or liver disease, advanced age, immunosuppression, and the presence of indwelling vascular lines [8].

The clinical presentation of fungal endocarditis is nonspecific and often indistinguishable from bacterial endocarditis, presenting significant diagnostic challenges. Common symptoms include fever, chills, malaise, dyspnea, cough, pleuritic chest pain, arthralgia, and myalgia. Physical examination findings may reveal a new or worsening cardiac murmur, signs of heart failure from valvular insufficiency, arrhythmias due to perivalvular abscesses, or extracardiac findings such as Janeway lesions, Osler nodes, Roth spots, and splinter hemorrhages. Systemic embolization may result in neurological, pulmonary, or organ dysfunction, further complicating diagnosis [9]. 

The low prevalence of fungal endocarditis compared to bacterial endocarditis contributes to its overall low pretest probability. Current Infectious Disease Society of America (IDSA) guidelines for IE recommend empiric antibiotic therapy, not antifungal agents, further delaying treatment for fungal IE until blood culture results are available [10]. This delay can allow for fungal proliferation, worsening patient outcomes.

Another significant diagnostic challenge is the low sensitivity of blood cultures in fungal endocarditis. A retrospective study involving over 270 patients with fungal endocarditis reported that only about 50% had positive blood cultures [2]. Negative blood cultures may result from the slow growth rate of fungal organisms or low fungal concentrations in the bloodstream, both common characteristics of fungal infections.

According to the 2023 Duke-International Society for Cardiovascular Infectious Diseases (Duke-ISCVID) criteria, a definitive diagnosis of IE requires at least one pathological criterion, two major criteria, one major and three minor criteria, or five minor criteria. Our patient fulfilled a pathological criterion: "Microorganisms identified in the context of clinical signs of active endocarditis in a vegetation” [11].

Historically, the diagnosis of *H. capsulatum* has relied on urine or serum antigen testing, serology, blood cultures, or direct tissue sampling. However, this case is unique not only because *H. capsulatum* IE is exceptionally rare but also because the diagnosis was confirmed using the Karius Test® despite a negative urine antigen, demonstrating its ability to overcome the limitations of traditional diagnostics.

A literature review reiterates the diagnostic challenges of traditional methods in identifying *Histoplasma* IE. A 2010 case report highlights a similar diagnostic challenge where the patient had negative *Histoplasma* antigen assays and negative blood cultures, with the diagnosis ultimately confirmed through aortic valve excision, microscopic examination, and tissue culture growth [12]. Similarly, a 2022 case report described a patient with negative *Histoplasma* urine antigen testing but a diagnosis confirmed via positive serology and histology [13].

There is also increasing evidence supporting the utility of the Karius Test® in diagnosing IE, particularly in cases involving fastidious or blood culture-negative pathogens. For example, case reports have documented its role in identifying pathogens such as *Coxiella burnetii*, *Streptococcus gordonii*, *Bartonella rochalimae*, *Aspergillus*, and *Anaerococcus hydrogenalis* [14-18]. Notably, a 2024 case report demonstrated the use of the Karius Test® to diagnose disseminated histoplasmosis involving mitral valve endocarditis [19].

Although the Karius Test® offers a novel, rapid approach to broad pathogen detection through microbial cell-free DNA sequencing, several important limitations must be considered. The test cannot differentiate between active infection, colonization, or transient DNAemia, which may lead to false-positive results if not interpreted carefully in the clinical context. Additionally, the Karius Test® does not provide antimicrobial susceptibility information, limiting its ability to fully guide targeted therapy. Cost and limited availability further constrain its widespread clinical application. Therefore, while the Karius Test® represents a valuable adjunct to traditional diagnostic methods, its findings should always be interpreted alongside clinical presentation, imaging, and conventional laboratory results to avoid misdiagnosis or inappropriate management.

This case report is also limited by its single-patient nature, which restricts generalizability. Diagnostic uncertainty remains, as pathogen identification relies on microbial cell-free DNA sequencing without histopathologic confirmation from the prosthetic valve. Additionally, the lack of long-term follow-up limits assessment of recurrence risk. Future research should include prospective studies or multi-center case series to better evaluate the role of advanced diagnostics, such as cell-free DNA sequencing, in the management of culture-negative endocarditis and to inform standardized clinical pathways. 

It is crucial for physicians to maintain a high index of suspicion for fungal endocarditis, particularly in patient populations at increased risk, such as those who are immunocompromised, use intravenous drugs, or have prosthetic valves. Early consultation with an infectious disease specialist is also essential in the management of IE. While the IDSA guidelines do not recommend empiric antifungal therapy for suspected IE, decisions should be made on a case-by-case basis, especially since delays in diagnosis can occur due to the challenges in identifying fungal causes of IE. Fungal endocarditis is a life-threatening condition with reported mortality rates as high as 72% [2]. *H. capsulatum*, although an exceedingly rare cause of IE, can result in devastating sequelae similar to bacterial endocarditis and should be considered in patients from endemic areas.

We encourage clinicians to consider the use of adjunctive diagnostic tools, such as the Karius Test®, in cases where conventional testing is inconclusive and rapid pathogen identification is critical for guiding timely treatment, such as in this case. In particular, for patients with suspected culture-negative endocarditis or other deep-seated infections, next-generation sequencing of microbial cell-free DNA can offer valuable diagnostic insights that might otherwise be missed with standard methods. While these technologies are not without limitations, as mentioned previously, and should be interpreted in the appropriate clinical context, their integration into diagnostic workflows has the potential to improve patient outcomes by facilitating earlier targeted therapy, minimizing unnecessary broad-spectrum antimicrobial use, and informing critical management decisions.

## Conclusions

This case highlights the diagnostic challenges of *H. capsulatum *prosthetic valve endocarditis and reinforces the importance of considering fungal pathogens in culture-negative IE, particularly in patients with prosthetic valves, immunocompromised status, or endemic exposure risk factors. Traditional diagnostic methods, such as blood cultures and urine antigen testing, may yield false-negative results, leading to delays that can increase the risk of complications, the need for surgical intervention, or mortality. The use of cfDNA sequencing, exemplified by the Karius Test®, offered rapid pathogen identification and guided timely antifungal therapy, supporting its role as a valuable adjunct when conventional diagnostics fail, as demonstrated by our patient's clinical and radiographic improvement on follow-up. However, limitations of microbial cfDNA testing, including cost, availability, and limited validation across all clinical settings, must be recognized, and results should be interpreted within the broader clinical context. Early multidisciplinary team involvement is essential to optimize management in complex cases of prosthetic valve endocarditis. Moving forward, microbial cfDNA sequencing could be algorithmically incorporated after initial standard testing is negative, particularly in high-risk populations. Future prospective and multicenter studies are needed to evaluate the cost-effectiveness, clinical utility, and outcomes impact of integrating diagnostics like microbial cfDNA sequencing into standard care pathways for culture-negative endocarditis. 
